# Major cardiovascular events and death in parents of children with type 1 diabetes: a register-based matched cohort study in Sweden

**DOI:** 10.1007/s00125-024-06200-w

**Published:** 2024-06-26

**Authors:** Beatrice Kennedy, Mona-Lisa Wernroth, Gorav Batra, Ulf Hammar, Cecilia Linroth, Annika Grönberg, Liisa Byberg, Tove Fall

**Affiliations:** 1 Molecular Epidemiology, Department of Medical Sciences, Uppsala University, Uppsala, Sweden; 2 SciLifeLab, Uppsala University, Uppsala, Sweden; 3 Uppsala Clinical Research Center, Uppsala University, Uppsala, Sweden; 4 Cardiology, Department of Medical Sciences, Uppsala University, Uppsala, Sweden; 5 Paediatric Inflammation, Metabolism and Child Health Research, Department of Women’s and Children’s Health, Uppsala University, Uppsala, Sweden; 6 Medical Epidemiology, Department of Surgical Sciences, Uppsala University, Uppsala, Sweden

**Keywords:** Children, Cohort study, Death, Epidemiology, Ischaemic heart disease, Major cardiovascular events, Parents, Sweden, Type 1 diabetes

## Abstract

**Aims/hypothesis:**

Parenting a child with type 1 diabetes has been associated with stress-related symptoms. This study aimed to elucidate the potential impact on parental risk of major cardiovascular events (MCE) and death.

**Methods:**

In this register-based study, we included the parents of 18,871 children, born 1987–2020 and diagnosed with type 1 diabetes in Sweden at <18 years. The median parental age at the child's diagnosis was 39.0 and 41.0 years for mothers and fathers, respectively. The cohort also encompassed 714,970 population-based matched parental control participants and 12,497 parental siblings. Cox proportional hazard regression models were employed to investigate the associations between having a child with type 1 diabetes and incident MCE and all-cause death, and, as secondary outcomes, acute coronary syndrome and ischaemic heart disease (IHD). We adjusted for potential confounders including parental type 1 diabetes and country of birth.

**Results:**

During follow-up (median 12 years, range 0–35), we detected no associations between parenting a child with type 1 diabetes and MCE in mothers (adjusted HR [aHR] 1.02; 95% CI 0.90, 1.15) or in fathers (aHR 1.01; 95% CI 0.94, 1.08). We noted an increased hazard of IHD in exposed mothers (aHR 1.21; 95% CI 1.05, 1.41) with no corresponding signal in fathers (aHR 0.97; 95% CI 0.89, 1.05). Parental sibling analysis did not confirm the association in exposed mothers (aHR 1.01; 95% CI 0.73, 1.41). We further observed a slightly increased hazard of all-cause death in exposed fathers (aHR 1.09; 95% CI 1.01, 1.18), with a similar but non-significant estimate noted in exposed mothers (aHR 1.07; 95% CI 0.96, 1.20). The estimates from the sibling analyses of all-cause death in fathers and mothers were 1.12 (95% CI 0.90, 1.38) and 0.73 (95% CI 0.55, 0.96), respectively.

**Conclusions/interpretation:**

Having a child diagnosed with type 1 diabetes in Sweden was not associated with MCE, but possibly with all-cause mortality. Further studies are needed to disentangle potential underlying mechanisms, and to investigate parental health outcomes across the full lifespan.

**Graphical Abstract:**

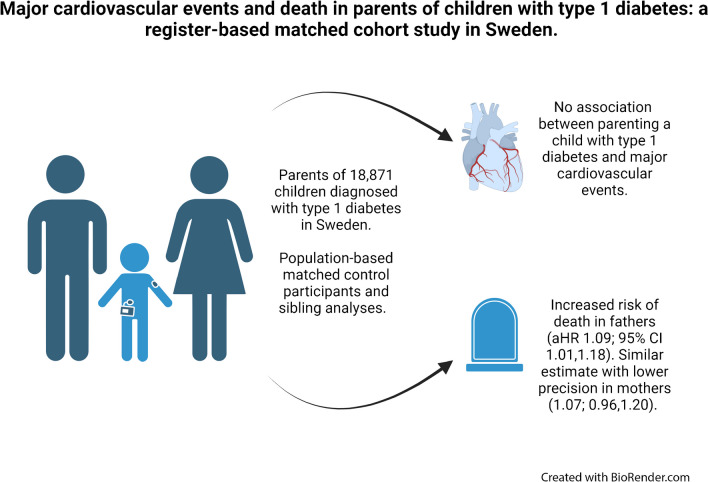

**Supplementary Information:**

The online version contains peer-reviewed and unedited supplementary material available at 10.1007/s00125-024-06200-w.


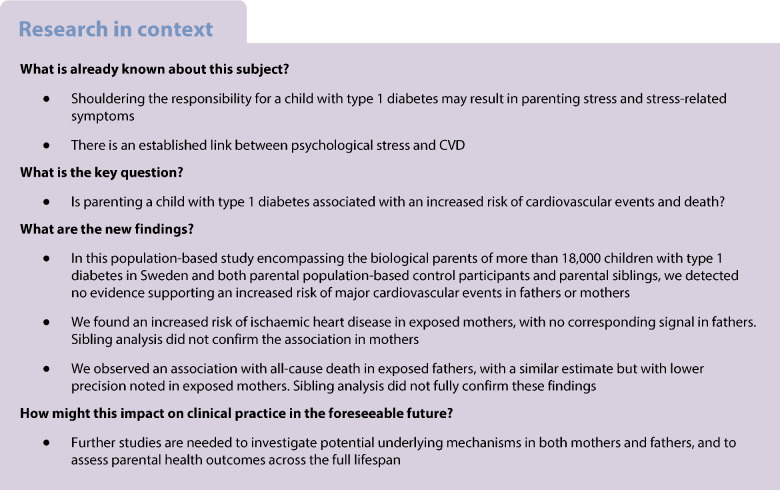


## Introduction

The overarching aims of type 1 diabetes treatment in childhood are to enable a high quality of life, and to minimise the risks of hypoglycaemic and diabetic ketoacidosis events, while also concurrently mitigating the long-term risk of microvascular and macrovascular complications [1]. For parents of younger children with type 1 diabetes, this translates to a need to administer appropriate doses of insulin several times a day, and to continuously monitor body glucose levels, dietary intake and physical activity level. Other parental challenges include managing the potential dire impact of, for example, common infections, the necessary and continual changes of the treatment regimen as the child grows, and the undulating insulin resistance encountered when the child enters puberty. Parents also need to ensure adequate diabetes care in preschool and school settings, and further encourage child self-efficacy and independence in disease management in older children [2]. In summary, having a child with type 1 diabetes may result in high levels of parenting stress. Elucidating the potential health consequences of such stress may help strengthen future parental support strategies.

Previous qualitative or questionnaire-based cross-sectional studies have indicated that the parents of children with type 1 diabetes experience emotional distress around the time of diagnosis [3], but also that parents, and mothers in particular, face an elevated risk of developing stress-related symptoms at a later stage [4, 5]. A recent register-based Swedish study reported that parents of children with type 1 diabetes were more likely to receive a diagnosis of depression, anxiety, or a stress-related disorder, and to be prescribed antidepressants or anxiolytics, than parents of children without type 1 diabetes [6]. Having a child with a chronic medical condition in Sweden, including type 1 diabetes, is further associated with an increase in parental sick leave and an elevated maternal risk of early retirement due to sickness or disability [7].

A vast body of knowledge links psychological stress to CVD [8, 9], with stress-related disorders associated with CVD independently of psychiatric comorbidities in sibling-based comparisons [10]. Recent register-based Nordic studies further indicated that exposure to severe parental stressors, such as the death of a child or having a child diagnosed with cancer, may influence the parental risk of ischaemic heart disease (IHD) including acute myocardial infarction (AMI) and haemorrhagic and ischaemic stroke [11–13]. However, we are not aware of any research that has investigated the potential parental cardiovascular health consequences of being the parent of a child with type 1 diabetes.

We therefore aimed to assess the risk of major cardiovascular events (MCE) and all-cause death in parents of children with type 1 diabetes as compared to population-based matched control participants and parental siblings. To this end, we have performed a register-based matched cohort study based on prospectively and objectively collected data from national population, health and quality registers in Sweden.

## Methods

### Study population

The source population of this study comprised parents in Sweden with biological children born between 1987 and 2020. The study population was based on register linkages across (1) national population registers held by Statistics Sweden: the Total Population Register, the Multi-Generation Register and the Longitudinal Integration Database for Health Insurance and Labour Market Studies (LISA) [14–16]; (2) national health registers held by the National Board of Health and Welfare: the National Patient Register (NPR), the Swedish Prescribed Drug Register and the Cause of Death Register [17–19]; and (3) the national quality Child Diabetes Register (Swediabkids) in Sweden, founded in 2000 [20]. The linkage was enabled by the unique personal identification numbers assigned to all residents of Sweden at birth or immigration. Only pseudonymised data were delivered to the research group. This study was approved by the Swedish Ethical Review Authority (DNR 2020-02206 with addendums 2020-04497 and 2021-01956).

### Main exposure, population-based control participants and parental siblings

Our main exposure was to be the biological parent of a child with type 1 diabetes. We included the biological parents of all children born between 1987 and 2020 who were diagnosed with type 1 diabetes before their 18th birthday in Sweden. Child type 1 diabetes was defined as an inpatient diagnosis in the NPR from 1987 and onwards (for all ICD codes used in this project, please see the electronic supplementary material [ESM] Methods) [17], and/or a prescription of insulin (the Anatomical Therapeutic Chemical code [ATC] A10A) in the Swedish Prescribed Drug Resister (initiated in July 2005), and/or a diagnosis of type 1 diabetes in Swediabkids (ESM Methods and ESM Tables 1 and 2). The index date was defined as the date of first entry in any of these registers. The parents of the children with type 1 diabetes were identified through the Multi-Generation Register.

For each exposed parent, we randomly selected 20 population-based parental control individuals from the underlying population using the Total Population Register. The control individuals were matched on parental sex and year of birth, and year of birth of their biological child. Furthermore, we extracted information on all biological siblings of the exposed parents, identified through the Multi-Generation Register, who had biological children of their own who had not been diagnosed with type 1 diabetes.

After exclusions (see detailed information in ESM Fig. 1a-c), we included 36,812 parents of children with type 1 diabetes, 714,970 population-based parental control individuals, and 12,497 parental siblings in our analyses. With this approach, the study population can be seen as representative of the overall Swedish population of parents during the study period.

### Main outcomes

An incident MCE comprised our main composite outcome. An MCE was defined as an inpatient main diagnosis in the NPR, or a main cause of death recorded in the Cause of Death Register, of an AMI, an ischaemic stroke, a haemorrhagic stroke, or any cardiovascular death (CV death). We further analysed incident AMI, ischaemic stroke and haemorrhagic stroke separately. As secondary outcomes, we investigated acute coronary syndrome (ACS; AMI or unstable angina) and IHD (AMI, unstable angina or stable coronary disease) defined by an inpatient main diagnosis in the NPR or a main cause of death recorded in the Cause of Death Register. All-cause mortality was defined as death by any cause in the Cause of Death Register.

### Covariates

Information on parental and child baseline comorbidity status at index date was obtained from the NPR. Parental type 1 diabetes was defined as a diagnosis of type 1 diabetes (main or secondary inpatient diagnosis and/or main outpatient diagnosis) in the NPR, and/or a diagnosis of type 1 diabetes in Swediabkids. Parental and child autoimmune conditions were defined as a main or secondary inpatient or outpatient diagnosis in the NPR of primary adrenal insufficiency, coeliac disease, atrophic gastritis or autoimmune thyroid illness.

Child neurodevelopmental comorbidities at index date were defined as a main or secondary inpatient or outpatient diagnosis of an autism spectrum disorder and/or of an attention-deficit/hyperactivity disorder, all recorded at ≥12 months of age.

Parental sociodemographic variables were obtained from annual excerpts from the Total Population Register and LISA. LISA includes sociodemographic information from 1990 and onwards. Parental variables comprised calendar year of birth, country of birth (categorised as Sweden, other Nordic countries and non-Nordic countries), highest education level (compulsory, secondary or university), annual household income, region of residence (Götaland, Svealand, Southern Norrland, Northern Norrland), population density of home municipality (number of inhabitants per km^2^), marital status (single, married or cohabitating with joint children), and the number of children aged <18 years in the household (0, 1, 2 or ≥3). We used covariate data from 31 December in the calendar year preceding the index year.

Using the DAGitty tool (https://www.dagitty.net/; version 3.1) [21], we drew a directed acyclic graph (DAG) prior to the analysis phase to identify potential confounders. The DAG is a graphical presentation of the theoretical framework and potential underlying assumed causal structures connecting exposures and covariates with the outcomes in our study (ESM Fig. 2). Child comorbidities and sociodemographic variables constituted potential effect modifiers, not included in our causal framework [22].

### Statistical analysis

We defined index date as date of diagnosis of type 1 diabetes in children to exposed parents, and we set the same date as index date in the matched parental control participants. If an exposed parent had more than one child diagnosed with type 1 diabetes during follow-up, the date of diagnosis of the first child was used as index date. Cox proportional hazard regression, stratified by matched set, with time since index date as timescale was used to calculate HR and 95% CIs for MCE and death, as well as for AMI, ischaemic stroke, and haemorrhagic stroke separately, comparing exposed parents to their population-based matched control participants. All analyses were performed in mothers and fathers separately. The dependence within individuals, due to sampling with replacement, was adjusted for using robust standard errors. Parents were censored at death, death of the child, emigration, or end of follow-up (31 December 2021), whichever came first. The multivariable-adjusted main model comprised the two time-updated variables parental age and parental type 1 diabetes status, as well as the following variables assessed at baseline: parental autoimmune comorbidities, parental country of birth, and population density of home municipality. As we did not exclude individuals with AMI, ischaemic stroke or haemorrhagic stroke, our main results reflect incident but not always first MCE.

The Schoenfeld residuals indicated that the proportional hazard assumption was violated for parental type 1 diabetes and parental country of birth and therefore an interaction with time since the index date was included for these two covariates.

We conducted parental sibling analysis to better account for residual confounding factors shared between siblings. We employed a Cox proportional regression model, stratified by sibling cluster, and used age as the timescale. We set the index date by parental age, i.e. the same age as the age of the exposed parental sibling at the time of the diagnosis of type 1 diabetes of their child. If a sibling had more than one child, the child closest in age to the cousin with type 1 diabetes was selected as the control child. Sibling clusters without any event do not contribute to the estimates in the sibling analysis, and we report informative sample size for the sibling analyses. The multivariable-adjusted sibling model comprised exposure status (having a child with type 1 diabetes [y/n]), index year, age of child at index date, population density of home municipality, parental country of birth, and time-updated parental type 1 diabetes status.

Potential effect modifiers were investigated by adding one multiplicative interaction at a time to the adjusted main model. The Wald test was used and a *p* value of <0.05 was considered statistically significant. We assessed the following baseline variables: age of parent, age of child, child neurodevelopmental and autoimmune comorbidities, and the parental sociodemographic variables: highest attained education level, annual household income, number of children aged <18 years in household, and marital status. Age of parent, age of child and annual household income were modelled using restricted cubic splines with four knots at the 5th, 35th, 65th and 95th percentiles. As sociodemographic variables were available in LISA from 1990 and onwards, sociodemographic analyses were only performed in parents with an index date in 1991 or later.

We conducted two sensitivity analyses. First, we assessed the MCE outcome in which we excluded all parents diagnosed with an AMI , ischaemic stroke or haemorrhagic stroke before the index date. Second, we conducted an analysis in which we only included parents and children born in Sweden, with a potentially more complete coverage of parental and child type 1 diabetes date of diagnoses and comorbidities.

We further explored if any association between the exposure and the main outcomes varied by time since index. To investigate the potential impact of having more than one child with type 1 diabetes, we also added, to the main model, a time-updated variable that indicated that an additional child had been diagnosed with type 1 diabetes (only the second child diagnosed with type 1 diabetes was included as very few parents had three or more children with type 1 diabetes).

As we detected elevated hazards for all-cause death in parents of children with type 1 diabetes as compared to parental control participants, we further performed a post hoc analysis exploring if we could detect increased hazards for deaths due to cancers or external causes of morbidity and mortality (including suicide, accidents, environmental exposures and homicides). These causes of death comprised the second and third most common causes of death in Sweden for individuals aged 30–74 during 1997–2022 (the most common was CVD death). We also categorised external causes of morbidity and mortality as suicide and other external causes, respectively. This subanalysis was only performed in parents with an index year in 1997 or later when ICD-10 codes were used. We employed the same multivariable adjustments in the post hoc analysis as in the main analyses.

We performed secondary analyses investigating ACS and IHD, using the same variables as in the multivariable-adjusted main model. We further conducted the same two secondary sensitivity analyses as for our main outcomes. Lastly, we conducted sibling analysis on IHD.

All analyses were conducted using R software (version 4.2.3, March 2023) [23].

## Results

Baseline characteristics for parents of children with type 1 diabetes and their population-based matched parental control participants are presented in Table 1. Median age at index date was 39.0 years for mothers and 41.0 years for fathers. Type 1 diabetes was six times more prevalent in parents of children diagnosed with type 1 diabetes (3.6% of mothers and 5.4% of fathers) than in the control participants (0.6% and 0.9%, respectively). Other autoimmune conditions, mainly coeliac disease and autoimmune thyroid disease, were also more commonly observed. We further noted that parents of children diagnosed with type 1 diabetes were more often born in Sweden, but were less often married and also less often had three or more children. Other socioeconomic and residential characteristics were similar across the groups.

**Table 1:** Baseline characteristics of mothers and fathers of children with type 1 diabetes (exposed) and their population-based matched control participants (unexposed)

Characteristic	Mothers exposed (*N*=18,597)	Mothers unexposed (*N*=361,370)	Fathers exposed (*N*=18,215)	Fathers unexposed (*N*=353,600)
Age (years) at index date, median (IQR)	39.0 (34.0–44.0)	39.0 (34.0–43.0)	41.0 (36.0–46.0)	41.0 (36.0–46.0)
Index year				
1987–1990	97 (0.5)	1,879 (0.5)	97 (0.5)	1,863 (0.5)
1991–2000	2918 (15.7)	56,797 (15.7)	2851 (15.7)	55,236 (15.6)
2001–2010	7271 (39.1)	141,353 (39.1)	7131 (39.1)	138,274 (39.1)
2011–2020	8311 (44.7)	161,341 (44.6)	8136 (44.7)	158,227 (44.7)
Type 1 diabetes^a^	663 (3.6)	2074 (0.6)	979 (5.4)	3121 (0.9)
Primary adrenal insufficiency^a^	10 (0.1)	97 (0.0)	14 (0.1)	86 (0.0)
Coeliac disease^a^	153 (0.8)	1317 (0.4)	57 (0.3)	478 (0.1)
Atrophic gastritis^a^	8 (0.0)	119 (0.0)	5 (0.0)	66 (0.0)
Autoimmune thyroid illness^a^	714 (3.8)	8942 (2.5)	136 (0.7)	1068 (0.3)
Country of birth				
Sweden	16,182 (87.0)	284,916 (78.8)	15,848 (87.0)	279,546 (79.1)
Nordic countries	479 (2.6)	8408 (2.3)	398 (2.2)	7649 (2.2)
Non-Nordic countries	1936 (10.4)	68,046 (18.8)	1969 (10.8)	66,405 (18.8)
Highest attained education level^b, c^				
Compulsory	1938 (10.4)	42,342 (11.7)	2455 (13.5)	52,288 (14.8)
Secondary	8748 (47.0)	161,865 (44.8)	9521 (52.3)	174,772 (49.4)
University	7682 (41.3)	150,416 (41.6)	6023 (33.1)	121,106 (34.2)
Annual household income quintile^b^^, c^				
1 (Lowest)	3360 (18.1)	72,226 (20.0)	3213 (17.6)	70,753 (20.0)
2	3683 (19.8)	71,918 (19.9)	3662 (20.1)	70,306 (19.9)
3	4011 (21.6)	71,613 (19.8)	3916 (21.5)	70,060 (19.8)
4	3811 (20.5)	71,780 (19.9)	3756 (20.6)	70,218 (19.9)
5 (Highest)	3635 (19.5)	71,954 (19.9)	3571 (19.6)	70,400 (19.9)
Marital status^b^				
Married	10,121 (54.4)	205,234 (56.8)	10,132 (55.6)	205,209 (58.0)
Cohabiting	4709 (25.3)	82,465 (22.8)	4656 (25.6)	81,713 (23.1)
Single	3670 (19.7)	71,788 (19.9)	3330 (18.3)	64,815 (18.3)
Number of children aged <18 years in the household^b^				
0	593 (3.2)	10,999 (3.0)	2582 (14.2)	51,652 (14.6)
1	3931 (21.1)	69,398 (19.2)	3263 (17.9)	57,335 (16.2)
2	9157 (49.2)	172,688 (47.8)	8122 (44.6)	151,385 (42.8)
≥3	4819 (25.9)	106,406 (29.4)	4151 (22.8)	91,365 (25.8)
Region of residence^b^				
Götaland	9101 (48.9)	173,463 (48.0)	8892 (48.8)	169,776 (48.0)
Svealand	7072 (38.0)	145,401 (40.2)	6920 (38.0)	141,755 (40.1)
Southern Norrland	1402 (7.5)	23,698 (6.6)	1378 (7.6)	23,443 (6.6)
Northern Norrland	1022 (5.5)	18,808 (5.2)	1025 (5.6)	18,626 (5.3)
Population density of home municipality^d^, median (IQR)	78.5 (29.2–240.0)	85.7 (31.8–416.7)	78.7 (29.2–252.9)	85.0 (31.7–415.3)

Data are *n* (%) unless otherwise stated

^a^ Assessed at index date

^b^ Assessed 31 December in the calendar year preceding the index year

^c^ Data missing for parents with an index year in 1987–1990. The variable highest attained education level also has missingness for later index years. The column percentages for these variables therefore do not add up to 100

^d^ Number of inhabitants per km^2^

During follow-up (median 12 years, range 0–35 years), type 1 diabetes was diagnosed in an additional 477 parents of children with type 1 diabetes, resulting in a prevalence of type 1 diabetes at end of follow-up of 4.6% in the exposed mothers and 6.9% in the exposed fathers. The prevalence in the matched control participants rose to 0.9% in mothers and 1.4% in fathers.

The median age of type 1 diabetes diagnosis in children was 9.2 years (IQR 5.4–12.7; Table 2). Children diagnosed with type 1 diabetes (*n*=18,871) were more often boys, more often born in Sweden, and had a higher prevalence of autoimmune conditions at index date than children of the parental control participants. Neurodevelopmental disorders were also slightly more common.

**Table 2:** Baseline characteristics of the children diagnosed with type 1 diabetes and of the children of the population-based parental control participants

Characteristic	Children diagnosed with type 1 diabetes (*N*=18,871)	Children of population-based parental control participants (*N*=714,970)
Age (years) at index date, median (IQR)	9.2 (5.4–12.7)	9.1 (5.4–12.6)
Boys	10,266 (54.4)	367,608 (51.4)
Country of birth		
Sweden	18,214 (96.5)	676,633 (94.6)
Nordic countries	69 (0.4)	2949 (0.4)
Non-Nordic countries	588 (3.1)	35,375 (4.9)
Autoimmune comorbidities^a^	410 (2.2)	4963 (0.7)
Neurodevelopmental comorbidities^b^	420 (2.2)	12,974 (1.8)

Data are *n* (%) unless otherwise stated

Comorbidities were assessed at the index date

^a^ Primary adrenal insufficiency, coeliac disease, atrophic gastritis and autoimmune thyroid illness

^b^ Autism spectrum disorders and attention-deficit/hyperactivity disorders

### Major cardiovascular events and all-cause death

**Fig. 1:**
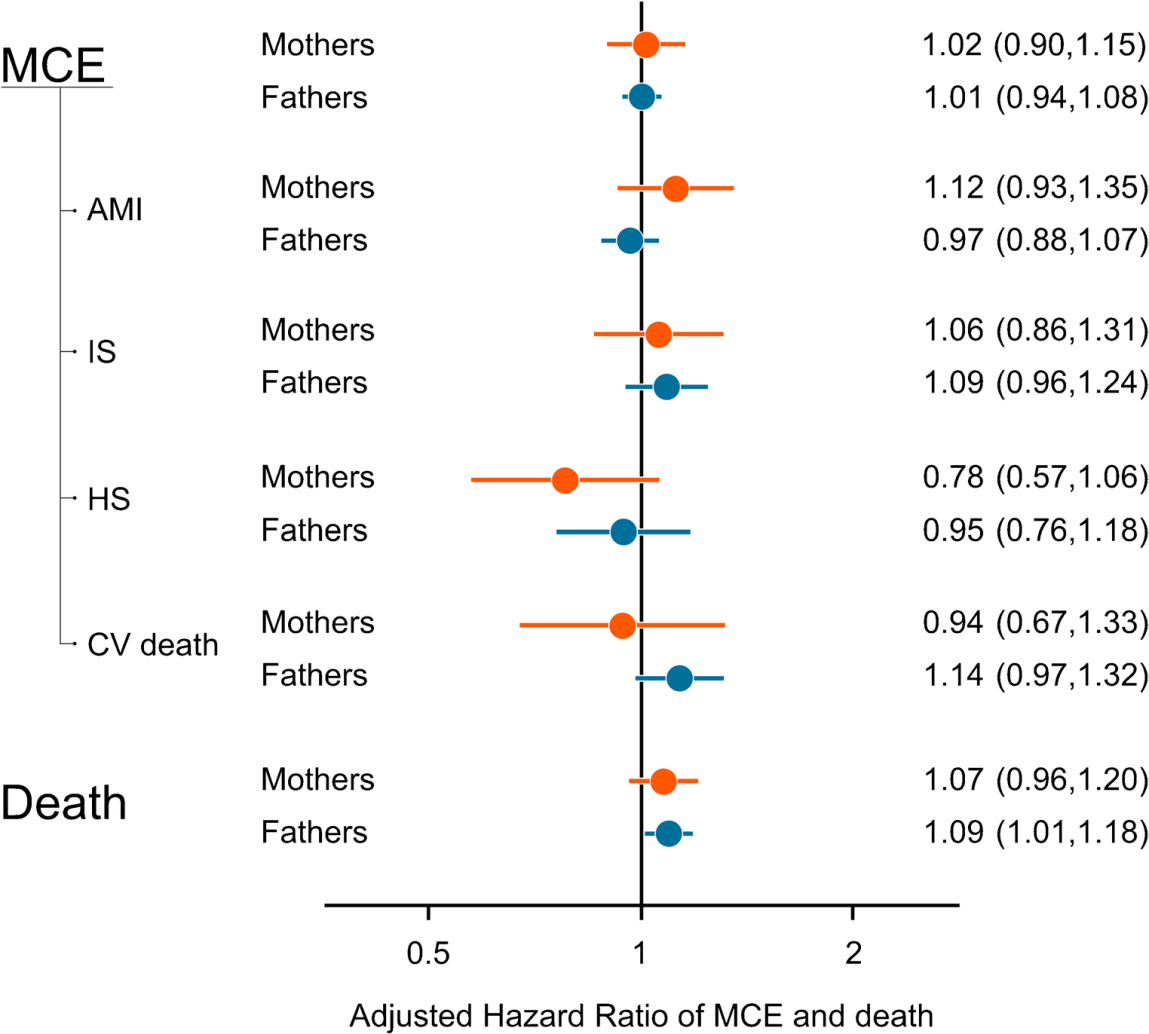
aHRs and 95% CIs for MCE (AMI, ischaemic stroke [IS], haemorrhagic stroke [HS] or CV death) and all-cause death in mothers and fathers of children with type 1 diabetes (exposed) as compared to population-based matched parental control individuals (unexposed). HRs are adjusted for the following parental variables: age (time-updated), type 1 diabetes (time-updated), autoimmune comorbidities, country of birth, and population density of home municipality

During follow-up, the incidence rate of MCE was 107 and 334 per 100 000 person-years in mothers and fathers of children with type 1 diabetes, respectively, with incidence rates of 98 and 330 observed in the maternal and paternal control participants. We detected no association between parenting a child with type 1 diabetes and MCE in mothers (adjusted HR [aHR] 1.02; 95% CI 0.90, 1.15) or in fathers (aHR 1.01; 95% CI 0.94, 1.08; Fig. 1 and ESM Table 3). Also, we could not discern any associations with AMI, ischaemic stroke, or haemorrhagic stroke when these outcomes were analysed separately. The elevated crude HR of CV death in fathers (1.21; 95% CI 1.04, 1.40) was attenuated after adjustments (aHR 1.14; 95% CI 0.97, 1.32). No corresponding signal was noted in mothers (aHR 0.94; 95% CI 0.67, 1.33). The sensitivity analysis of MCE where we assessed only first incident events yielded similar findings as the main model in mothers and fathers (ESM Tables 4 and 5).

The mortality rate was 127 and 281 per 100 000 person-years in mothers and fathers of children with type 1 diabetes, respectively, and 114 and 243 in maternal and paternal control individuals. We observed an association with all-cause death in fathers (aHR 1.09; 95% CI 1.01, 1.18). In mothers, the point estimate was similar but with lower precision (aHR 1.07; 95% CI 0.96, 1.20). In a post hoc analysis, the point estimates for death due to cancer (aHR 1.09; 95% CI 0.95, 1.25) and external causes of morbidity and mortality (aHR 1.08; 95% CI 0.89, 1.30) in fathers mirrored the point estimates noted for all-cause death and CV death, but with lower precision (ESM Table 6). The point estimates for mothers were somewhat lower (aHR 0.99 [95% CI 0.85, 1.17] and aHR 1.03 [95% CI 0.76, 1.41] for cancer and external causes of morbidity and mortality, respectively). Detailed estimates for suicide and other external causes in parents with an index year in 1997–2020 are available in ESM Table 6.

Baseline characteristics of the mothers and fathers, and their children, included in the sibling analysis of MCE and death are available in ESM Tables 7–10. In the sibling analysis, the estimate for all-cause death in fathers resembled that in the main analysis but with wider confidence intervals (aHR 1.12; 95% CI 0.90, 1.38; ESM Table 11). In contrast, the elevated estimate from the main analysis for all-cause death in mothers was not reproduced (aHR 0.73; 95% CI 0.55, 0.96).

In the sensitivity analysis including only parents and children born in Sweden (ESM Table 12), the HRs for all-cause death were attenuated (aHR 1.04 [95% CI 0.92, 1.17] and 1.05 [0.97, 1.14] in mothers and fathers, respectively).

We found no evidence of effect modification of age of parent, age of child, parental socioeconomic circumstances or child comorbidities on MCE or death (ESM Table 13). Further, we noted no interactions between time since index and the main outcomes (ESM Fig. 3).

During follow-up, 921 mothers and 899 fathers had an additional child diagnosed with type 1 diabetes. We observed higher point estimates for MCE (aHR 1.59; 95% CI 0.98, 2.59) and all-cause death (aHR 1.25; 95% CI 0.76, 2.07) in those mothers, as compared with population-based matched control participants, than in the main analyses. In contrast, the point estimates of MCE (aHR 0.84; 95% CI 0.59, 1.19) and all-cause death (aHR 0.78; 95% CI 0.52, 1.17) noted in those fathers were lower.

### Acute coronary syndrome and ischaemic heart disease

**Fig. 2:**
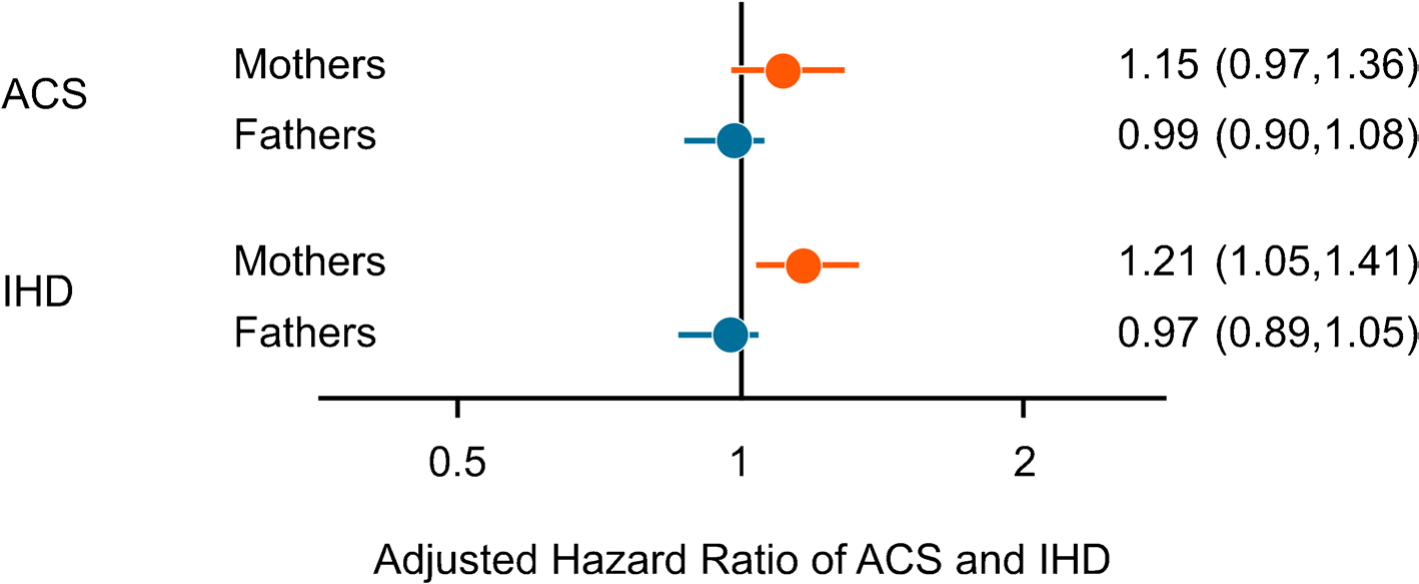
aHRs and 95% CIs for ACS (including AMI and unstable angina) and IHD (including AMI, unstable angina and stable coronary artery disease) in mothers and fathers of children with type 1 diabetes (exposed) as compared to population-based matched parental control individuals (unexposed). HRs are adjusted for the following parental variables: age (time-updated), type 1 diabetes (time-updated), autoimmune comorbidities, country of birth, and population density of home municipality

The incidence rates for ACS were 58 and 216 per 100,000 person-years in mothers and fathers of children with type 1 diabetes, respectively, with incidence rates of 45 and 221 observed in the maternal and paternal control individuals. Incidence rates for IHD were 77 and 270 in exposed mothers and fathers, respectively, and 58 and 278 in control participants. We observed elevated point estimates for ACS and IHD in exposed mothers (aHR 1.15 [95% CI 0.97, 1.36] and aHR 1.21 [95% CI 1.05, 1,41] for ACS and IHD, respectively), although the association with ACS lacked statistical significance (Fig. 2 and ESM Table 14). We noted no association with ACS or IHD in fathers (aHR 0.99 [95% CI 0.90, 1.08] and aHR 0.97 [95% CI 0.89, 1.05], respectively). In sensitivity analyses, we noted similar estimates of IHD in parents born in Sweden, as well as when we excluded parents with a record of IHD before the index date (ESM Table 15–16). Sibling analysis did not confirm the increased hazard of IHD in mothers (aHR 1.01; 95% CI 0.73, 1.41; ESM Tables 17–19).

## Discussion

In this large population-based matched cohort study encompassing the biological parents of more than 18,000 children diagnosed with type 1 diabetes in Sweden, we could not detect any associations between having a child diagnosed with type 1 diabetes before the age of 18 and incident parental MCE. We observed a slightly elevated HR for all-cause death in fathers, with a similar estimate observed in mothers, with the latter not supported in sibling analysis. We noted no effect modification of parental socioeconomic circumstances, child comorbidities, or time since the type 1 diagnosis of the child.

Strengths of this study include the population-based study design, the use of prospectively and objectively collected high-quality register data, and the essentially complete follow-up. Some limitations apply. First, our findings mainly reflect incident MCE and death in middle adulthood with a low overall incidence of the outcomes, more so in women. We cannot exclude the possibility that parenting a child with type 1 diabetes may entail a differential risk of cardiovascular morbidity or mortality later in life. Further, even though we included the biological parents of all children born 1987–2020 diagnosed with type 1 diabetes in Sweden, we had limited power when assessing different causes of death or the impact of having an additional child diagnosed with type 1 diabetes. Second, information on parental lifestyle behaviours including physical activity levels, smoking habits and sleep patterns is not available in national health registers, and we could not investigate if such factors mediated or modified the associations. Third, we could not fully ascertain the familial living arrangements at index or during follow-up. We found no evidence of effect modification of parental cohabitation status on our main outcomes, but our register-based data sources did not enable us to assess, for instance, how much a divorced parent with a child with type 1 diabetes cohabitated with the child or was involved in the day-to-day care. Fourth, during our study period, child healthcare visits and hospital admissions have been heavily subsidised or fully free of charge in Sweden. Prescriptions of insulin, diabetes supplies and diabetes medical devices including continuous glucose monitoring systems and insulin pumps have all been provided free of charge. Moreover, parents of children with type 1 diabetes have been eligible for monthly parental childcare allowance, and have also received at least partial compensation for loss of income when caring for a sick child. Taken together, these factors may have served to alleviate some of the potential financial strain and subsequent psychological stress that could affect parents of a child with a chronic illness. The overall generalisability of our findings may therefore not extend to other countries with higher medical costs for children and different social security reimbursement systems. Fifth, we did not have access to procedural codes on, for example, percutaneous coronary intervention (PCI) or coronary artery bypass graft (CABG) surgery. However, our secondary outcomes included detailed inpatient data on the coronary artery disease diagnoses that constituted the main indications for PCI and CABG during our study period [24–26]. Lastly, cardiovascular mortality has decreased markedly in Sweden over the last few decades [27], which has been attributed to improved primary and secondary cardiovascular prevention programmes as well as better event survival rates due to improved emergency treatments. During the same time period, overall mortality rates in both men and women in middle adulthood have also decreased in Sweden. The external validity of our results is therefore limited to other countries with similar time trends in CVD and mortality rates.

We observed an association with higher IHD rates in mothers of children with type 1 diabetes, but not significantly so with ACS or AMI. As our cohort was mainly comprised of parents in middle adulthood, our findings could indicate that the mothers caring for a child with type 1 diabetes included in our study are at an increased risk of early development of coronary artery disease which had not yet resulted in a major cardiovascular event. However, our IHD findings were not confirmed in sibling analysis, potentially due to residual genetic confounding and/or a low number of events in sibling pairs, and our results should therefore be interpreted with caution.

Our findings only partly aligned with previous Nordic register-based studies reporting overall increased risks of CVD in parents exposed to loss of a child [11] or exposed to having a child diagnosed with cancer [13]. Some of the differences may be due to methodological dissimilarities across the studies. For example, the increased risk of IHD noted in bereaved parents occurred mainly after loss of a child aged <1 or >18 [11], and an increased risk of CVD in parents of children diagnosed with cancer was mainly observed in parents of children diagnosed after the age of 18. Here, we assessed parents of children diagnosed with type 1 diabetes at age 6 months to 17 years, although our follow-up continued beyond those ages. In correspondence with our results, more pronounced associations with IHD were noted in mothers than in fathers in both studies [11, 13].

We observed a modestly increased hazard of all-cause death in fathers of children with type 1 diabetes as compared to control individuals, with a similar but less precise estimate noted in mothers. Similar estimates were also noted for the three most common causes of death (CV death, cancer death, and death due to external causes of morbidity and mortality) in fathers, with no corresponding patterns in mothers. The sibling analyses also indicated an increased all-cause hazard of death in fathers but not in mothers, indicating that our findings in mothers may be partly due to residual confounding. When we restricted our cohort to parents and children born in Sweden, with potentially higher accuracy of, for example, the date of diagnosis of type 1 diabetes in the parents, the estimates for all-cause mortality were somewhat attenuated in both fathers and mothers. The low precision of our all-cause death estimate in mothers, as well as in the separate analyses of cause of death in fathers, can be attributed to the low rates of deaths during follow-up, especially in mothers.

If our findings indicate true elevated hazards of IHD in mothers, and of death in parents of children with type 1 diabetes, potential underlying mechanisms may include unfavourable changes in lifestyle behaviours, disadvantageous socioeconomic consequences including reduced income and labour market attachment, and/or neuroendocrine and immune system modulation due to parenting stress, all contributing to increased risk of somatic and/or psychiatric disease development and long-term adverse health effects. Further studies are needed to disentangle any such associations, which could differ between mothers and fathers.

In conclusion, we could not detect any association between parenting a child with type 1 diabetes and MCE. However, we noted an association with IHD in mothers, not confirmed in sibling analysis, and slightly increased hazards for all-cause death. Further studies with longer follow-up are required to investigate potential underlying mechanisms and the risk of morbidity and death across the full lifespan.

## Supplementary Information

Below is the link to the electronic supplementary material.

Supplementary file1 (PDF 850 KB)

## Data Availability

Restrictions apply to the availability of these data, which were used under license and ethical approval and are not publicly available. Data are, however, available from the authors upon reasonable request and with written permission of the Swedish Ethical Review Authority, subject to legal contracts regarding the General Data Protection Regulation (GDPR) and Personal Data Processing Agreements between Uppsala University and the receiving research entity.

## Abbreviations

ACS: Acute coronary syndrome
aHR: Adjusted HR
AMI: Acute myocardial infarction
CV death: Cardiovascular death
IHD: Ischaemic heart disease
LISA: Longitudinal Integration Database for Health Insurance and Labour Market Studies
MCE: Major cardiovascular event
NPR: National Patient Register
