# Supplementary material for: Major cardiovascular events and death in parents of children with type 1 diabetes: a register-based matched cohort study in Sweden
Source: Diabetologia. 2024 Jun 26;67(9):1828–37. doi: 10.1007/s00125-024-06200-w (PMC11410917; doi:10.1007/s00125-024-06200-w)
Supplement: Supplementary file 1 — Supplementary file1 (PDF 850 KB) [file 125_2024_6200_MOESM1_ESM.pdf]

METHODS

ICD-9 and ICD-10 codes

Child and parental type 1 diabetes was defined by the International Classification of Diseases, 9th revision code [ICD-9] 250 or the International Classification of Diseases, 10th revision code [ICD-10] E10.

A major cardiovascular event (MCE) was defined as an in-patient main diagnosis in the NPR or a main cause of death recorded in the Cause of Death register of 1) acute myocardial infarction (AMI; ICD-10 I21; ICD-9 410), 2) ischaemic stroke (ICD-10 I63; ICD-9 434), 3) haemorrhagic stroke (ICD-10 I60-I62; ICD-9 430, 431, 432) or, 4) any cardiovascular death as the main cause of death recorded in the Cause of Death Register (ICD-10 I00-I99; ICD-9 39x-45x).

Acute coronary syndrome (ACS) was defined as an in-patient main diagnosis in the NPR or a main cause of death recorded in the Cause of Death register of AMI or unstable angina (ICD-10 121 and I20.0; ICD-9 410 and 411.1). Ischemic heart disease (IHD) was defined as an in-patient main diagnosis in the NPR or a main cause of death recorded in the Cause of Death register of AMI, unstable angina, or stable coronary artery disease (ICD-10 I20-I25; ICD-9 410-414).

Parental and child autoimmune conditions were defined as a main or secondary inpatient or outpatient diagnosis in the NPR of a) primary adrenal insufficiency (ICD-10 E27.1, ICD-9 255E), b) celiac disease (ICD-10 K90.0, ICD-9 579A), c) atrophic gastritis (ICD-10 K29.4, ICD-9 535B), or d) autoimmune thyroid illness ([hypothyroidism: ICD-10 E03.8, E03.9, E06.3, ICD-9 244X], [hyperthyroidism: ICD-10 E05.0, E05.9, E06.2, E06.9; ICD-9 245C, 242A]).

Child neurodevelopmental comorbidities at index date were defined as a main or secondary inpatient or outpatient diagnosis of an autism spectrum disorder (ASD; ICD-10 F84, ICD-9 299) and/or of an attention-deficit/hyperactivity disorder (ADHD; ICD-10 F90, ICD-9 314), all recorded at ≥12 months of age.

In post-hoc analyses, we explored deaths due to cancers (ICD-9 140-239, ICD-10 C00-D48) or external causes of morbidity and mortality (ICD-9 E800-E999, ICD-10 V01-Y98). For parents with an index year in 1997-2020, we categorized external causes of morbidity and mortality as suicide (ICD-10 X60-X84 and Y10-Y34) and other external causes, respectively.

Ascertainment of the exposure

We used three different data sources to ascertain a child type 1 diabetes diagnosis (at age <18 years):

1. During our study period, the national recommendation was to initially hospitalize all children at diagnosis, and they should therefore have an inpatient diagnosis of type 1 diabetes in the *National Patient Register*. This register was the only available data source for 1987-2000, although children alive and residing in Sweden in subsequent years may also have records in the other two registers at <18 years. Children who were diagnosed abroad will not have a recorded inpatient diagnosis of type 1 diabetes unless hospitalized in Sweden for type 1 diabetes adverse events or other diseases at a later stage.
2. The Swedish national quality child diabetes register *Swediabkids* was founded in 2000, and in 2007 all child diabetes clinics across the country were enrolled to report data, including specific types of diabetes. The overall coverage of all children with type 1 diabetes in Swediabkids was estimated to be 92.7% in 2019, with regional differences in coverage across Sweden ranging from 77.5% to 98.7% [1]. As Swediabkids includes information on the date of diagnosis, even if it was not in Sweden or before the register was initiated, this register may help increase coverage and improve detail on the index date for children not diagnosed in Sweden.
3. *The Swedish Prescribed Drug Register* was initiated in 2005 and contains information on all dispensed prescriptions of insulin. Although insulin may also be prescribed to children with other rare types of diabetes, this register helps increase coverage of children not diagnosed with type 1 diabetes in Sweden and who attend a child diabetes clinic that does not fully submit data to Swediabkids.

Out of the 18,871 children used to define our exposed parental group, 17,480 (92.6%) had records in all three registers, 924 (4.8%) in two registers, and 467 (2.5%) children had records in only one register (ESM Table 2). To validate the type 1 diabetes diagnosis in these 476 children, we used outpatient data from the National Patient Register (which holds information on specialist outpatient visits from 2001 and onwards) and diagnoses from the Cause of Death Register. Of the 467, 158 children had a diagnosis of type 1 diabetes recorded from an outpatient visit in the National Patient Register, and 6 had a record of type 1 diabetes in the Cause of Death Register.

In summary, out of the 18,871 children in our study population, 303 (1.6%) had a record in only one of the three main data sources and their type 1 diabetes diagnosis could not be validated in out-patient or death data. We have therefore performed an extra sensitivity analysis on our main outcomes where we exclude the parents of these 303 children. We observed nearly identical estimates as in the main analysis (ESM Table 3).

ESM Table 1. Cross-tabulation summarizing the three registers used to identify the 18,871 children with type 1 diabetes included in our study population.

Type 1 diabetes inpatient diagnosis in the NPR	Prescription of insulin in the SPDR	Type 1 diabetes diagnosis in Swediabkids	Frequency	Percent
1	1	1	17,480	92.6
**Sum of children identified from all 3 registers**			**17,480**	**92.6**
1	1	0	725	3.8
0	1	1	196	1.0
1	0	1	3	0.0
**Sum of children identified from 2 registers**			**924**	**4.8**
1	0	0	150	0.8
0	0	1	27	0.1
0	1	0	290	1.5
**Sum of children identified from 1 register**			**467**	**2.4**

NPR: The National Patient Register

SPDR: The Swedish Prescribed Drug Register

ESM Table 2. Sensitivity analysis excluding the parents of the 303 children with type 1 diabetes only identified in one of the three source registers and with no record of type 1 diabetes from an out-patient visit in the National Patient Register or in the Cause of Death Register. Adjusted hazard ratios (aHRs) with 95% CIs for major cardiovascular events (MCE; encompassing acute myocardial infarction, ischaemic or haemorrhagic stroke, and cardiovascular death) and all-cause death in mothers and fathers of children with type 1 diabetes (exposed), as compared to parental full biological siblings with children without type 1 diabetes.

	**Mothers**	**Fathers**
**Outcome**	**aHR (95% CI)**	**aHR (95% CI)**
MCE	1.02 (0.90, 1.16)	1.00 (0.93, 1.07)
Death	1.07 (0.95, 1.20)	1.09 (1.01, 1.18)

HRs adjusted for parental age (time-updated), type 1 diabetes status (time-updated), autoimmune comorbidities, country of birth and population density of home municipality.

ESM Table 3. Crude and adjusted hazard ratios (cHRs and aHRs) with 95% CIs for major cardiovascular events (MCE; acute myocardial infarction, ischaemic or haemorrhagic stroke, and cardiovascular death) and all-cause death in mothers and fathers of children with type 1 diabetes (exposed), as compared to population-based maternal and paternal controls (unexposed).

**Outcome**	**Mothers**				**Fathers**			
	**Number of events in exposed**	**Number of events in unexposed**	**cHR (95% CI)**	**aHR (95% CI)**	**Number of events in exposed**	**Number of events in unexposed**	**cHR (95% CI)**	**aHR (95% CI)**
MCE	264	4,652	1.08 (0.95,1.21)	1.02 (0.90,1.15)	787	14,887	1.00 (0.94,1.08)	1.01 (0.94,1.08)
AMI	118	1,841	1.22 (1.01,1.46)	1.12 (0.93,1.35)	432	8,543	0.96 (0.87,1.05)	0.97 (0.88,1.07)
IS	96	1,629	1.13 (0.92,1.38)	1.06 (0.86,1.31)	235	4,013	1.11 (0.97,1.26)	1.09 (0.96,1.24)
HS	43	1,049	0.78 (0.58,1.05)	0.78 (0.57,1.06)	82	1,654	0.95 (0.76,1.18)	0.95 (0.76,1.18)
CV Death	38	686	1.04 (0.75,1.43)	0.94 (0.67,1.33)	172	2,725	1.21 (1.04,1.40)	1.14 (0.97,1.32)
Death	315	5,447	1.10 (0.98,1.22)	1.07 (0.96,1.20)	676	11,158	1.15 (1.06,1.24)	1.09 (1.01,1.18)

HRs adjusted for parental age (time-updated), type 1 diabetes status (time-updated), autoimmune comorbidities, country of birth and population density of home municipality.

AMI: Acute myocardial infarction
IS: Ischaemic stroke
HS: Haemorrhagic stroke
CV Death: Cardiovascular death

ESM Table 4. Number of parents of children with type 1 diabetes (exposed) and population-based parental controls (unexposed) diagnosed with an acute myocardial infarction, an ischaemic stroke or a haemorrhagic stroke before index date.

	**Mothers Exposed**	**Mothers Unexposed**	**Fathers Exposed**	**Fathers Unexposed**
	n = 18,597	n = 361,370	n = 18,215	n = 353,600
Event before index (%)				
AMI	17 (0.1)	202 (0.1)	107 (0.6)	1,523 (0.4)
IS	11 (0.1)	311 (0.1)	42 (0.2)	595 (0.2)
HS	13 (0.1)	375 (0.1)	22 (0.1)	477 (0.1)
**Total**	**40 (0.2)**	**877 (0.2)**	**165 (0.9)**	**2 538 (0.7)**

AMI: Acute myocardial infarction

IS: ischaemic stroke

HS: Haemorrhagic stroke

ESM Table 5. Sensitivity analysis excluding parents with an acute myocardial infarction, an ischaemic or a haemorrhagic stroke before the index date. Adjusted hazard ratios (aHRs) with 95% CIs for first incident major cardiovascular event (MCE) in mothers and fathers of children with type 1 diabetes (exposed), as compared to population-based maternal and paternal controls (unexposed).

	**Mothers**			**Fathers**		
**Outcome**	**Exposed N events /N parents**	**Unexposed N events /N parents**	**aHR (95% CI)**	**Exposed N events /N parents**	**Unexposed N events /N parents**	**aHR (95% CI)**
MCE	260/ 18,557	4,544/ 359,730	1.03 (0.91,1.17)	735/ 18,050	14,115/ 348,013	0.98 (0.91,1.05)

HRs adjusted for parental age (time-updated), type 1 diabetes status (time-updated), autoimmune comorbidities, country of birth and population density of home municipality.

ESM Table 6. Post-hoc analysis of deaths due to cancer or external causes of morbidity and mortality in mothers and fathers of children with type 1 diabetes (exposed), as compared to population-based maternal and paternal controls (unexposed). The additional analysis where external causes are categorized as suicide and other external causes (including accidents, environmental exposures, and homicides) was conducted only in parents with an index year in 1997-2020.

**Cause of death**	**Mothers**				**Fathers**			
	**Number of events in exposed**	**Number of events in unexposed**	**cHR (95% CI)**	**aHR (95% CI)**	**Number of events in exposed**	**Number of events in unexposed**	**cHR (95% CI)**	**aHR (95% CI)**
Cancer	155	3,057	0.96 (0.82,1.13)	0.99 (0.85,1.17)	218	3,817	1.07 (0.94,1.23)	1.09 (0.95,1.25)
External causes of morbidity and mortality	41	736	1.05 (0.77,1.44)	1.03 (0.76,1.41)	118	2,032	1.11 (0.92,1.33)	1.08 (0.89,1.30)
- Suicide	23	394	1.11 (0.73,1.67)	1.08 (0.71,1.65)	57	879	1.25 (0.96,1.63)	1.20 (0.92,1.58)
- Other external causes	12	235	0.98 (0.55,173)	0.93 (0.53,1.63)	39	853	0.87 (0.63,1.19)	0.84 (0.61,1.15)

cHR: Crude hazard ratio

aHR: Adjusted hazard ratio

HRs adjusted for parental age (time-updated), type 1 diabetes status (time-updated), autoimmune comorbidities, country of birth and population density of home municipality.

ESM Table 7. Baseline characteristics of the mothers and fathers of children with type 1 diabetes (exposed) and their parental siblings included in the sibling analysis of major cardiovascular events.

	**Mothers exposed**	**Maternal siblings**	**Fathers exposed**	**Paternal siblings**
N	123	175	304	420
Year of birth (%)				
1940-1949	1 (0.8)	1 (0.6)	9 (3.0)	11 (2.6)
1950-1959	21 (17.1)	37 (21.1)	93 (30.6)	157 (37.4)
1960-1969	75 (61.0)	92 (52.6)	162 (53.3)	198 (47.1)
1970-1979	24 (19.5)	41 (23.4)	38 (12.5)	49 (11.7)
1980-1989	2 (1.6)	4 (2.3)	2 (0.7)	5 (1.2)
Age at child's birth, median (IQR), years	29.0 (26.0:32.0)	29.0 (26.0:32.0)	33.0 (29.0:36.0)	33.0 (29.0:36.0)
Age at index date, median (IQR), years	39.0 (35.0:43.0)	39.0 (35.0:42.0)	42.0 (38.0:46.0)	42.0 (38.0:46.0)
Index year (%)				
1987-1990	1 (0.8)	1 (0.6)	1 (0.3)	7 (1.7)
1991-2000	34 (27.6)	63 (36.0)	73 (24.0)	128 (30.5)
2001-2010	69 (56.1)	83 (47.4)	179 (58.9)	207 (49.3)
2011-2020	19 (15.4)	28 (16.0)	51 (16.8)	78 (18.6)
Type 1 diabetes[a] (%)	12 (9.8)	8 (4.6)	33 (10.9)	13 (3.1)
Other autoimmune disease [a, b] (%)	7 (5.7)	4 (2.3)	4 (1.3)	4 (1.0)
Country of birth, No (%)				
Sweden	120 (97.6)	168 (96.0)	294 (96.7)	406 (96.7)
Other Nordic countries	2 (1.6)	4 (2.3)	6 (2.0)	6 (1.4)
Non-Nordic countries	1 (0.8)	3 (1.7)	4 (1.3)	8 (1.9)
Highest education [c, d] (%)				
Compulsory	17 (13.8)	30 (17.1)	46 (15.1)	68 (16.2)
Secondary	67 (54.5)	98 (56.0)	174 (57.2)	239 (56.9)
University	38 (30.9)	45 (25.7)	82 (27.0)	104 (24.8)
Household income quintile[c, d] (%)				
1 (Lowest)	25 (20.3)	45 (25.7)	78 (25.7)	127 (30.2)

2	26 (21.1)	52 (29.7)	55 (18.1)	100 (23.8)
3	18 (14.6)	41 (23.4)	58 (19.1)	89 (21.2)
4	21 (17.1)	22 (12.6)	54 (17.8)	61 (14.5)
5 (Highest)	32 (26.0)	14 (8.0)	58 (19.1)	36 (8.6)
Marital status[c] (%)				
Married	64 (52.0)	90 (51.4)	172 (56.6)	239 (56.9)
Cohabiting	38 (30.9)	39 (22.3)	67 (22.0)	94 (22.4)
Single	20 (16.3)	45 (25.7)	64 (21.1)	80 (19.0)
Number of children in the household[c] (%)				
0	5 (4.1)	6 (3.4)	45 (14.8)	72 (17.1)
1	24 (19.5)	37 (21.1)	43 (14.1)	74 (17.6)
2	59 (48.0)	88 (50.3)	125 (41.1)	171 (40.7)
≥3	34 (27.6)	43 (24.6)	90 (29.6)	96 (22.9)
Region of residence[c] (%)				
Götaland	73 (59.3)	100 (57.1)	149 (49.0)	206 (49.0)
Svealand	32 (26.0)	51 (29.1)	102 (33.6)	138 (32.9)
Southern Norrland	9 (7.3)	10 (5.7)	37 (12.2)	46 (11.0)
Northern Norrland	9 (7.3)	14 (8.0)	16 (5.3)	30 (7.1)
Population density[c], median (IQR)	47.5 (23.7:103.2)	46.6 (19.4:98.8)	57.2 (22.9:127.4)	58.5 (24.1:129.6)

[a] Assessed at index date.

[b] Includes primary adrenal insufficiency, celiac disease, atrophic gastritis, and autoimmune thyroid illness.

[c] Assessed December 31 the calendar year preceding the index year.

[d] Data not available for parents with an index year in 1987-1990. The variable highest achieved education level has missingness for later index years as well. The column percentages for these variables do therefore not add up to 100.

ESM Table 8. Baseline characteristics of the children of the parents included in the sibling analysis of major cardiovascular events.

	**Children of exposed mothers**	**Children of maternal siblings**	**Children of exposed fathers**	**Children of paternal siblings**
	n = 123	n = 175	n = 304	n = 420
Boys (%)	67 (54.5)	95 (54.3)	182 (59.9)	225 (53.6)
Year of birth (%)				
1987-1990	32 (26.0)	57 (32.6)	88 (28.9)	126 (30.0)
1991-2000	74 (60.2)	89 (50.9)	163 (53.6)	219 (52.1)
2001-2010	15 (12.2)	29 (16.6)	49 (16.1)	68 (16.2)
2011-2020	2 (1.6)	0 (0.0)	4 (1.3)	7 (1.7)
Age at index date, median (IQR), years	10.9 (6.4:14.1)	9.9 (6.4:13.3)	9.8 (6.3:12.8)	9.1 (5.6:12.9)

ESM Table 9. Baseline characteristics of the mothers and fathers of children with type 1 diabetes (exposed) and their parental siblings included in the sibling analysis of all-cause death.

	Mothers exposed	Maternal siblings	Fathers exposed	Paternal siblings
N	144	193	228	313
Year of birth (%)				
1940-1949	1 (0.7)	3 (1.6)	9 (3.9)	11 (3.5)
1950-1959	33 (22.9)	40 (20.7)	75 (32.9)	119 (38.0)
1960-1969	79 (54.9)	106 (54.9)	115 (50.4)	141 (45.0)
1970-1979	29 (20.1)	39 (20.2)	25 (11.0)	39 (12.5)
1980-1989	2 (1.4)	5 (2.6)	4 (1.8)	3 (1.0)
Age at child's birth, median (IQR), years	30.0 (27.0:33.0)	30.0 (27.0:33.0)	32.0 (29.0:36.0)	32.0 (29.0:36.0)
Age at index date, median (IQR), years	40.0 (35.0:44.0)	39.0 (36.0:44.0)	42.0 (38.0:46.0)	41.0 (38.0:46.0)
Index year (%)				
1987-1990	1 (0.7)	5 (2.6)	2 (0.9)	6 (1.9)
1991-2000	46 (31.9)	57 (29.5)	65 (28.5)	97 (31.0)
2001-2010	74 (51.4)	93 (48.2)	128 (56.1)	165 (52.7)
2011-2020	23 (16.0)	38 (19.7)	33 (14.5)	45 (14.4)
Type 1 diabetes[a] (%)	7 (4.9)	7 (3.6)	25 (11.0)	14 (4.5)
Other autoimmune disease[a, b] (%)	2 (1.4)	9 (4.7)	2 (0.9)	4 (1.3)
Country of birth (%)				
Sweden	140 (97.2)	182 (94.3)	219 (96.1)	304 (97.1)
Other Nordic countries	1 (0.7)	2 (1.0)	7 (3.1)	6 (1.9)
Non-Nordic countries	3 (2.1)	9 (4.7)	2 (0.9)	3 (1.0)
Highest education[c, d] (%)				
Compulsory	24 (16.7)	26 (13.5)	53 (23.2)	72 (23.0)
Secondary	80 (55.6)	112 (58.0)	115 (50.4)	176 (56.2)
University	39 (27.1)	48 (24.9)	58 (25.4)	59 (18.8)
Household income quintile[c, d] (%)				
1 (Lowest)	35 (24.3)	54 (28.0)	72 (31.6)	103 (32.9)

2	30 (20.8)	46 (23.8)	31 (13.6)	92 (29.4)
3	34 (23.6)	34 (17.6)	50 (21.9)	53 (16.9)
4	25 (17.4)	36 (18.7)	34 (14.9)	32 (10.2)
5 (Highest)	19 (13.2)	18 (9.3)	39 (17.1)	27 (8.6)
Marital status[c] (%)				
Married	74 (51.4)	113 (58.5)	125 (54.8)	155 (49.5)
Cohabiting	35 (24.3)	37 (19.2)	42 (18.4)	75 (24.0)
Single	34 (23.6)	38 (19.7)	59 (25.9)	77 (24.6)
Number of children in the household[c] (%)				
0	4 (2.8)	5 (2.6)	37 (16.2)	73 (23.3)
1	33 (22.9)	61 (31.6)	31 (13.6)	58 (18.5)
2	73 (50.7)	81 (42.0)	90 (39.5)	109 (34.8)
≥3	33 (22.9)	41 (21.2)	68 (29.8)	67 (21.4)
Region of residence[c] (%)				
Götaland	72 (50.0)	99 (51.3)	119 (52.2)	149 (47.6)
Svealand	53 (36.8)	69 (35.8)	70 (30.7)	117 (37.4)
Southern Norrland	12 (8.3)	17 (8.8)	22 (9.6)	28 (8.9)
Northern Norrland	7 (4.9)	8 (4.1)	17 (7.5)	19 (6.1)
Population density[c], median (IQR)	61.7 (21.0:109.2)	70.1 (26.2:127.2)	66.9 (24.3:155.5)	59.5 (24.4:138.6)

[a] Assessed at index date.

[b] Includes primary adrenal insufficiency, celiac disease, atrophic gastritis, and autoimmune thyroid illness.

[c] Assessed December 31 the calendar year preceding the index year.

[d] Data not available for parents with an index year in 1987-1990. The variable highest achieved education level has missingness for later index years as well. The column percentages for these variables do therefore not add up to 100.

ESM Table 10. Baseline characteristics of the children of the parents included in the sibling analysis of all-cause death.

	**Children of exposed mothers**	**Children of maternal siblings**	**Children of exposed fathers**	**Children of paternal siblings**
	n = 144	n = 193	n = 228	n = 313
Boys (%)	78 (54.2)	91 (47.2)	137 (60.1)	167 (53.4)
Year of birth (%)				
1987-1990	41 (28.5)	55 (28.5)	77 (33.8)	107 (34.2)
1991-2000	79 (54.9)	111 (57.5)	114 (50.0)	161 (51.4)
2001-2010	23 (16.0)	26 (13.5)	34 (14.9)	44 (14.1)
2011-2020	1 (0.7)	1 (0.5)	3 (1.3)	1 (0.3)
Age at index date, median (IQR), years	9.9 (5.8:12.7)	10.4 (5.2:13.7)	9.9 (7.0:12.6)	9.1 (5.4:13.0)

ESM Table 11. Sibling analysis. Crude and adjusted hazard ratios (cHRs and aHRs) and 95% CIs for major cardiovascular events (MCE; encompassing acute myocardial infarction, ischaemic or haemorrhagic stroke, and cardiovascular death) and all-cause death in mothers and fathers of children with type 1 diabetes (exposed), as compared to parental full biological siblings with children without type 1 diabetes.

**Outcome**	**Mothers**				**Fathers**			
	**Number of events in exposed**	**Number of events in siblings**	**cHR (95% CI)**	**aHR (95% CI)**	**Number of events in exposed**	**Number of events in siblings**	**cHR (95% CI)**	**aHR (95% CI)**
MCE	48	79	0.79 (0.59,1.04)	0.76 (0.57,1.02)	141	190	1.00 (0.84,1.18)	0.88 (0.73,1.06)
Death	60	85	0.84 (0.66,1.08)	0.73 (0.55,0.96)	113	123	1.15 (0.94,1.40)	1.12 (0.90,1.38)

HR adjusted for index year, child’s age at index date, population density of home municipality, parental country of birth, and time-updated parental type 1 diabetes status.

ESM Table 12. Sensitivity analysis including only parents and children born in Sweden. Adjusted hazard ratios (aHRs) and 95% CIs for major cardiovascular events (MCE; acute myocardial infarction, ischaemic or haemorrhagic stroke, and cardiovascular death) and all-cause death in mothers and fathers of children with type 1 diabetes (exposed), as compared to population-based matched controls unexposed).

**Outcome**	**Mothers**			**Fathers**		
	**Exposed N events/N parents**	**Unexposed N events/N parents**	**aHR (95%CI)**	**Exposed N events/N parents**	**Unexposed N events/N parents**	**aHR (95%CI)**
MCE	230/16,095	3,764/283,144	1.02 (0.89,1.16)	686/15,777	11,526/278,019	1.02 (0.95,1.10)
Death	270/16,095	4,552/283,144	1.04 (0.92,1.17)	573/15,777	8,943/278,019	1.05 (0.97,1.14)

HRs adjusted for parental age (time-updated), type 1 diabetes status (time-updated), autoimmune comorbidities, country of birth and population density of home municipality.

ESM Table 13. Effect modification of the association between exposure (child diagnosed with type 1 diabetes) by parental and child age at index date, parental socioeconomic circumstances, and child comorbidities on the outcomes major cardiovascular events (MCE) and all-cause death.

	**p-values**			
	**Mothers**		**Fathers**	
**Effect modifier**	**MCE**	**Death**	**MCE**	**Death**
Age of child at index date	0.909	0.075	0.122	0.519
Age of parent at index date	0.673	0.074	0.622	0.832
Highest achieved education level	0.204	0.221	0.081	0.554
Annual household income	0.413	0.078	0.294	0.346
Marital status	0.245	0.774	0.308	0.543
Number of children in the household	0.120	0.586	0.158	0.502
Child autoimmune comorbidities[a]	0.872	0.199	0.251	0.282
Child neurodevelopmental comorbidities[b]	0.689	0.760	0.958	0.762

[a]Includes primary adrenal insufficiency, celiac disease, atrophic gastritis, and autoimmune thyroid illness
[b]Includes autism spectrum disorders and attention-deficit/hyperactivity disorders

ESM Table 14. Secondary analyses. Crude and adjusted hazard ratios (cHRs and aHRs) with 95% CIs for acute coronary syndrome (ACS; including acute myocardial infarction and unstable angina) and ischemic heart disease (IHD; including acute myocardial infarction, unstable angina, and stable coronary artery disease) in mothers and fathers of children with type 1 diabetes (exposed), as compared to population-based maternal and paternal controls (unexposed).

**Outcome**	**Mothers**				**Fathers**			
	**Number of events in exposed**	**Number of events in unexposed**	**cHR (95% CI)**	**aHR (95% CI)**	**Number of events in exposed**	**Number of events in unexposed**	**cHR (95% CI)**	**aHR (95% CI)**
ACS	143	2,133	1.27 (1.08,1.50)	1.15 (0.97,1.36)	512	10,023	0.98 (0.89,1.06)	0.99 (0.90,1.08)
IHD	191	2,751	1.32 (1.14,1.52)	1.21 (1.05,1.41)	636	12,557	0.96 (0.89,1.04)	0.97 (0.89,1.05)

HRs adjusted for parental age (time-updated), type 1 diabetes status (time-updated), autoimmune comorbidities, country of birth and population density of home municipality.

ESM Table 15. Secondary analysis. Sensitivity analysis including only parents and children born in Sweden. Adjusted hazard ratios (aHRs) and 95% CIs for acute coronary syndrome (ACS; including acute myocardial infarction and unstable angina) and ischemic heart disease (IHD; including acute myocardial infarction, unstable angina, and stable coronary artery disease) in mothers and fathers of children with type 1 diabetes (exposed), as compared to population-based matched controls (unexposed).

**Outcome**	**Mothers**			**Fathers**		
	**Exposed N events/N parents**	**Unexposed N events/N parents**	**aHR (95%CI)**	**Exposed N events/N parents**	**Unexposed N events/N parents**	**aHR (95%CI)**
ACS	126/16,095	1,673/283,144	1.19 (0.99,1.42)	431/15,777	7,423/278,019	0.99 (0.90,1.09)
IHD	168/16,095	2,123/283,144	1.25 (1.07,1.47)	538/15,777	9,260/278,019	0.98 (0.90,1.07)

HRs adjusted for parental age (time-updated), type 1 diabetes status (time-updated), autoimmune comorbidities, country of birth and population density of home municipality.

ESM Table 16. Secondary analysis. Sensitivity analysis excluding parents with an inpatient diagnosis of acute coronary syndrome (ACS; including acute myocardial infarction and unstable angina) or ischemic heart disease (IHD; including acute myocardial infarction, unstable angina, and stable coronary artery disease), respectively, before the index date. Adjusted hazard ratios (aHRs) with 95% CIs for first ACS or first IHD, in mothers and fathers of children with type 1 diabetes (exposed), as compared to population-based maternal and paternal controls (unexposed).

	**Mothers**			**Fathers**		
**Outcome**	**Exposed N events /N parents**	**Unexposed N events /N parents**	**aHR (95% CI)**	**Exposed N events /N parents**	**Unexposed N events /N parents**	**aHR (95% CI)**
ACS	136/18,575	2,088/360,714	1.14 (0.96,1.36)	471/18,089	9,452/349,416	0.95 (0.87,1.04)
IHD	179/18,566	2,666/360,404	1.20 (1.03,1.40)	569/18,053	11,511/348,180	0.93 (0.86,1.01)

HRs adjusted for parental age (time-updated), type 1 diabetes status (time-updated), autoimmune comorbidities, country of birth and population density of home municipality.

ESM Table 17. Secondary analysis. Baseline characteristics of the mothers and fathers of children with type 1 diabetes (exposed) and their parental siblings included in the sibling analysis of ischemic heart disease.

	**Mothers exposed**	**Maternal siblings**	**Fathers exposed**	**Paternal siblings**
N	93	129	248	348
Year of birth (%)				
1940-1949	1 (1.1)	0 (0.0)	7 (2.8)	11 (3.2)
1950-1959	18 (19.4)	26 (20.2)	76 (30.6)	133 (38.2)
1960-1969	59 (63.4)	75 (58.1)	135 (54.4)	163 (46.8)
1970-1979	14 (15.1)	28 (21.7)	28 (11.3)	38 (10.9)
1980-1989	1 (1.1)	0 (0.0)	2 (0.8)	3 (0.9)
Age at child's birth, median (IQR), years	29.0 (27.0:32.0)	29.0 (26.0:32.0)	33.0 (29.0:36.0)	33.0 (29.0:36.0)
Age at index date, median (IQR), years	40.0 (35.0:43.0)	39.0 (36.0:43.0)	42.5 (38.5:46.0)	42.0 (38.0:46.0)
Index year (%)				
1987-1990	0 (0.0)	1 (0.8)	0 (0.0)	4 (1.1)
1991-2000	25 (26.9)	48 (37.2)	61 (24.6)	113 (32.5)
2001-2010	60 (64.5)	61 (47.3)	146 (58.9)	171 (49.1)
2011-2020	8 (8.6)	19 (14.7)	41 (16.5)	60 (17.2)
Type 1 diabetes[a] (%)	11 (11.8)	8 (6.2)	36 (14.5)	15 (4.3)
Other autoimmune disease[a, b] (%)	3 (3.2)	3 (2.3)	2 (0.8)	3 (0.9)
Country of birth, No (%)				
Sweden	90 (96.8)	126 (97.7)	237 (95.6)	332 (95.4)
Other Nordic countries	3 (3.2)	3 (2.3)	6 (2.4)	6 (1.7)
Non-Nordic countries	0 (0.0)	0 (0.0)	5 (2.0)	10 (2.9)
Highest education[c, d] (%)				
Compulsory	11 (11.8)	18 (14.0)	40 (16.1)	60 (17.2)
Secondary	54 (58.1)	72 (55.8)	146 (58.9)	190 (54.6)
University	28 (30.1)	37 (28.7)	62 (25.0)	92 (26.4)

	**Mothers exposed**	**Maternal siblings**	**Fathers exposed**	**Paternal siblings**
Household income quintile[c, d] (%)				
1 (Lowest)	11 (11.8)	36 (27.9)	67 (27.0)	93 (26.7)
2	19 (20.4)	39 (30.2)	41 (16.5)	86 (24.7)
3	21 (22.6)	27 (20.9)	48 (19.4)	80 (23.0)
4	15 (16.1)	19 (14.7)	48 (19.4)	57 (16.4)
5 (Highest)	27 (29.0)	7 (5.4)	44 (17.7)	28 (8.0)
Marital status[c] (%)				
Married	54 (58.1)	69 (53.5)	138 (55.6)	203 (58.3)
Cohabiting	29 (31.2)	26 (20.2)	56 (22.6)	86 (24.7)
Single	10 (10.8)	33 (25.6)	54 (21.8)	55 (15.8)
Number of children in the household[c] (%)				
0	3 (3.2)	4 (3.1)	41 (16.5)	51 (14.7)
1	18 (19.4)	35 (27.1)	35 (14.1)	55 (15.8)
2	42 (45.2)	53 (41.1)	94 (37.9)	145 (41.7)
≥3	30 (32.3)	36 (27.9)	78 (31.5)	93 (26.7)
Region of residence[c] (%)				
Götaland	49 (52.7)	72 (55.8)	125 (50.4)	174 (50.0)
Svealand	34 (36.6)	38 (29.5)	81 (32.7)	108 (31.0)
Southern Norrland	4 (4.3)	4 (3.1)	27 (10.9)	37 (10.6)
Northern Norrland	6 (6.5)	15 (11.6)	15 (6.0)	29 (8.3)
Population density[c], median (IQR)	72.6 (26.2:106.4)	45.2 (18.3:94.1)	58.1 (20.0:123.4)	57.9 (19.8:128.7)

[a] Assessed at index date.

[b] Includes primary adrenal insufficiency, celiac disease, atrophic gastritis, and autoimmune thyroid illness.

[c] Assessed December 31 the calendar year preceding the index year.

[d] Data not available for parents with an index year in 1987-1990. The variable highest achieved education level has missingness for later index years as well. The column percentages for these variables do therefore not add up to 100.

ESM Table 18. Secondary analysis. Baseline characteristics of the children of the parents included in the sibling analysis of ischemic heart disease.

	Children of exposed mothers	Children of maternal siblings	Children of exposed fathers	Children of paternal siblings
N	93	129	248	348
Boys (%)	51 (54.8)	68 (52.7)	146 (58.9)	193 (55.5)
Year of birth (%)				
1987-1990	26 (28.0)	41 (31.8)	75 (30.2)	104 (29.9)
1991-2000	58 (62.4)	75 (58.1)	131 (52.8)	183 (52.6)
2001-2010	8 (8.6)	12 (9.3)	38 (15.3)	56 (16.1)
2011-2020	1 (1.1)	1 (0.8)	4 (1.6)	5 (1.4)
Age at index date, median (IQR), years	10.4 (6.4:13.5)	10.1 (6.3:13.5)	10.3 (6.6:13.2)	9.3 (5.9:12.8)

ESM Table 19. Secondary analysis. Sibling analysis. Crude and adjusted hazard ratios (cHRs and aHRs) and 95% CIs for ischemic heart disease (IHD; including acute myocardial infarction, unstable angina, and stable coronary artery disease) in mothers and fathers of children with type 1 diabetes (exposed), as compared to parental full biological siblings with children without type 1 diabetes.

**Outcome**	**Mothers**				**Fathers**			
	**Number of events in exposed**	**Number of events in siblings**	**cHR (95% CI)**	**aHR (95% CI)**	**Number of events in exposed**	**Number of events in siblings**	**cHR (95% CI)**	**aHR (95% CI)**
IHD	42	53	1.04 (0.76,1.41)	1.01 (0.73,1.41)	117	165	0.98 (0.82,1.18)	0.87 (0.70,1.09)

HR adjusted for index year, child's age at index date, population density of home municipality, parental country of birth, and time-updated parental type 1 diabetes status.

ESM Fig. 1. Flow charts of study populations and exclusions of a) parents of children with type 1 diabetes, b) matched population-based controls, and c) parental siblings.

a)

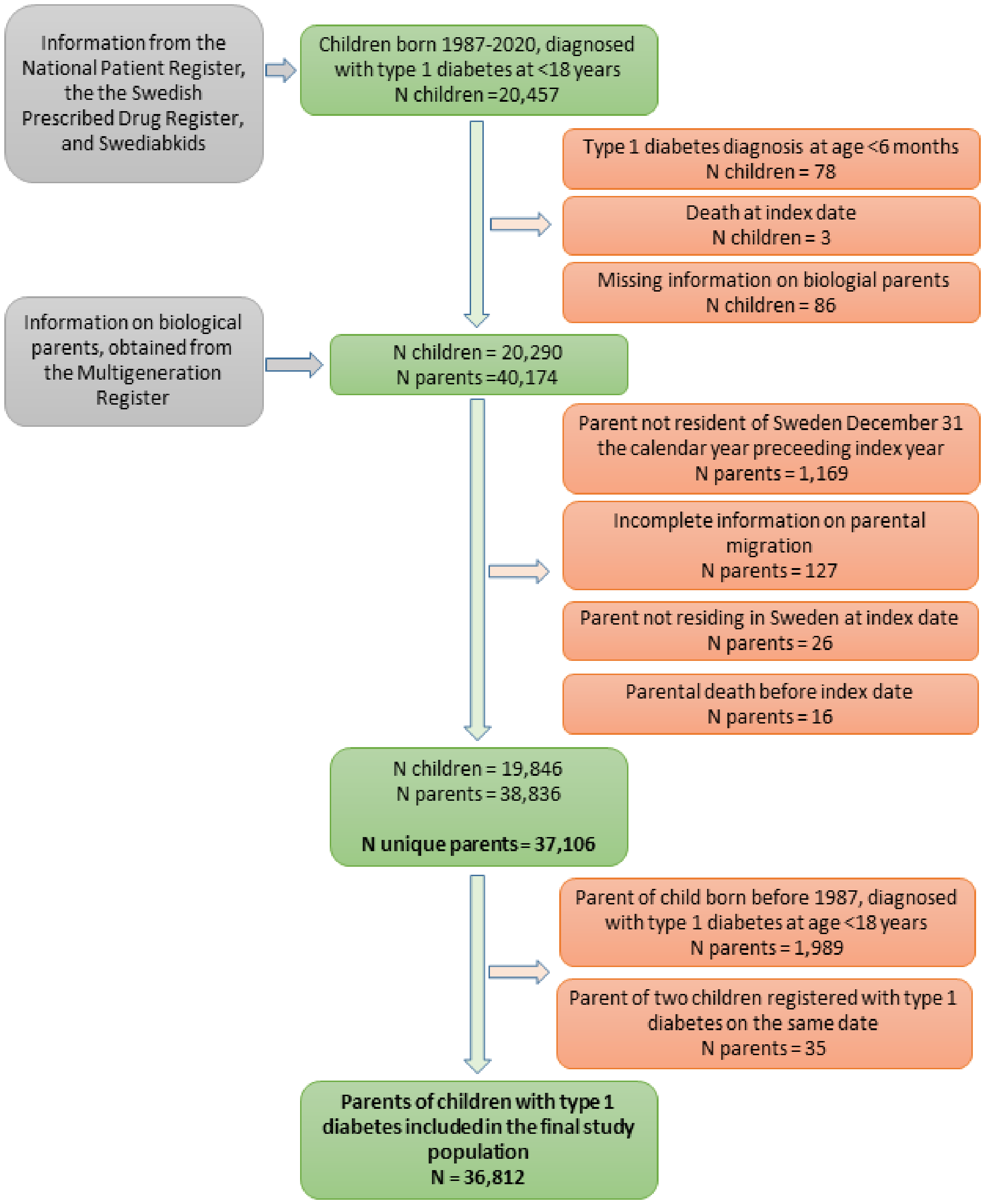

b)

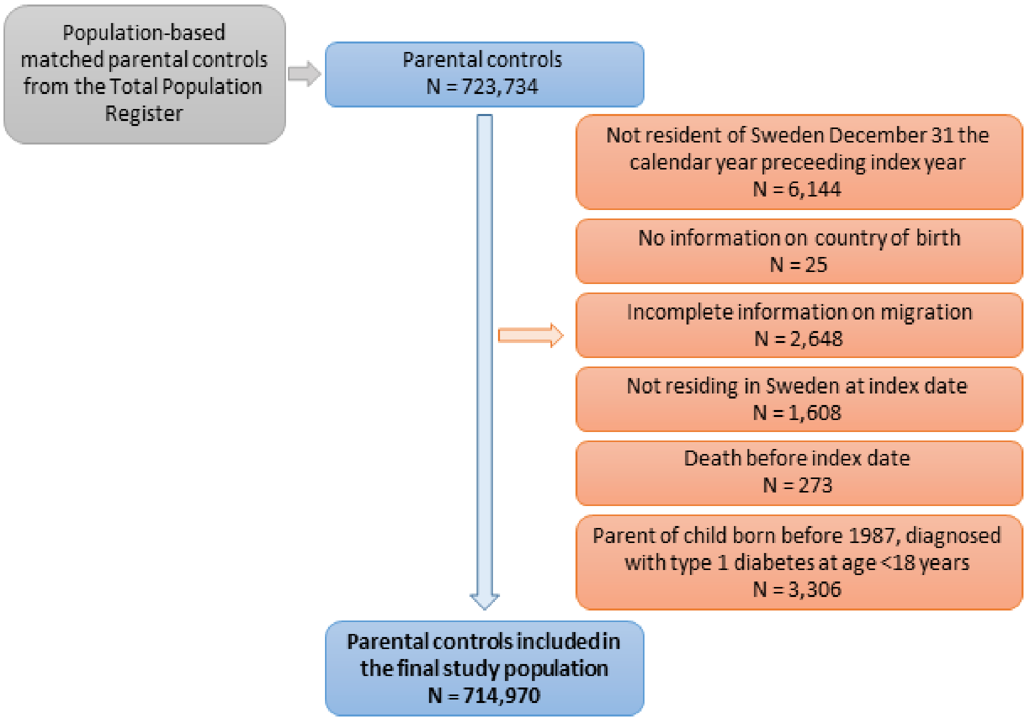
Population-based matched parental controls from the Total Population Register
Parental controls
N = 723,734
Not resident of Sweden December 31 the calendar year preceeding index year
N = 6,144
No information on country of birth
N = 25
Incomplete information on migration
N = 2,648
Not residing in Sweden at index date
N = 1,608
Death before index date
N = 273
Parent of child born before 1987, diagnosed with type 1 diabetes at age <18 years
N = 3,306
Parental controls included in the final study population
N = 714,970

c)

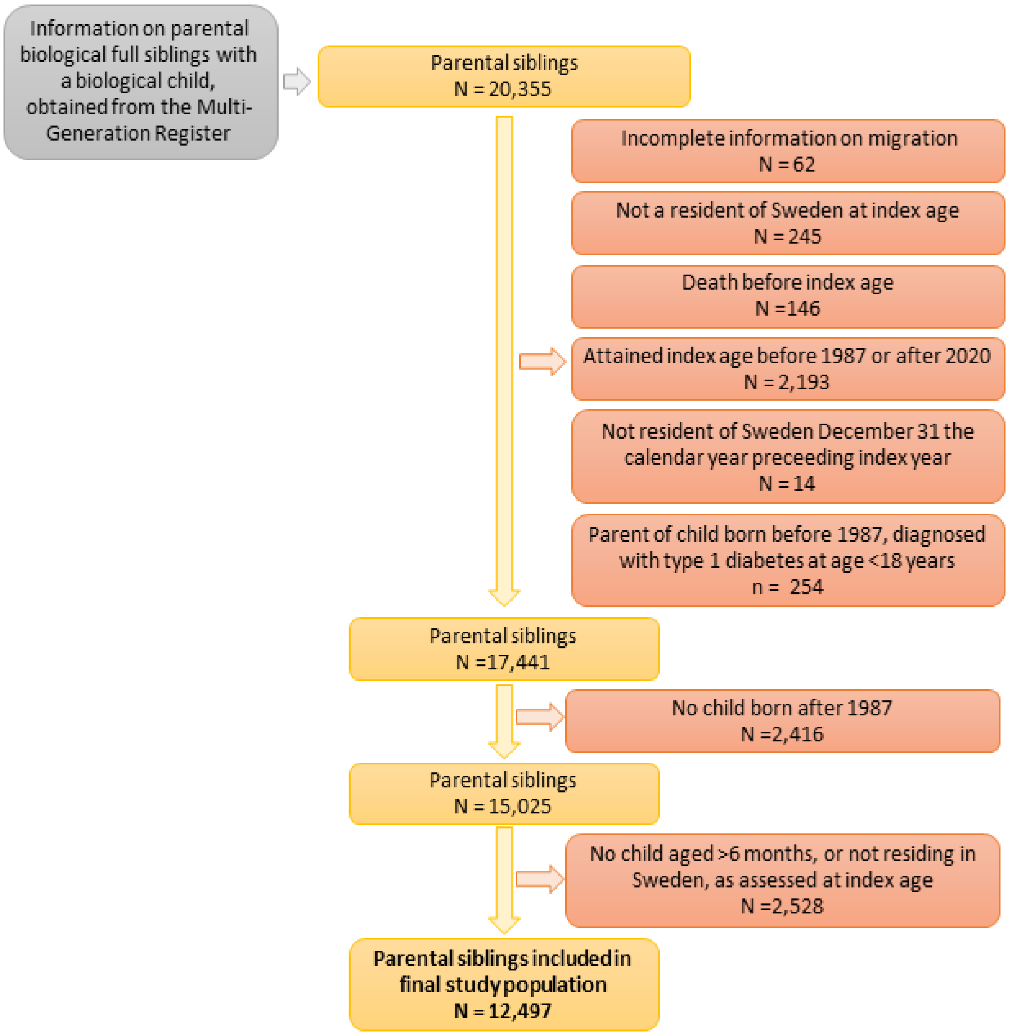
Information on parental biological full siblings with a biological child, obtained from the Multi-Generation Register
Parental siblings
N = 20,355
Incomplete information on migration
N = 62
Not a resident of Sweden at index age
N = 245
Death before index age
N =146
Attained index age before 1987 or after 2020
N = 2,193
Not resident of Sweden December 31 the calendar year preceeding index year
N = 14
Parent of child born before 1987, diagnosed with type 1 diabetes at age <18 years
n = 254
Parental siblings
N =17,441
No child born after 1987
N =2,416
Parental siblings
N = 15,025
No child aged >6 months, or not residing in Sweden, as assessed at index age
N =2,528
Parental siblings included in final study population
N = 12,497

ESM Fig. 2. Directed acyclic graph (DAG) of the theoretical framework.

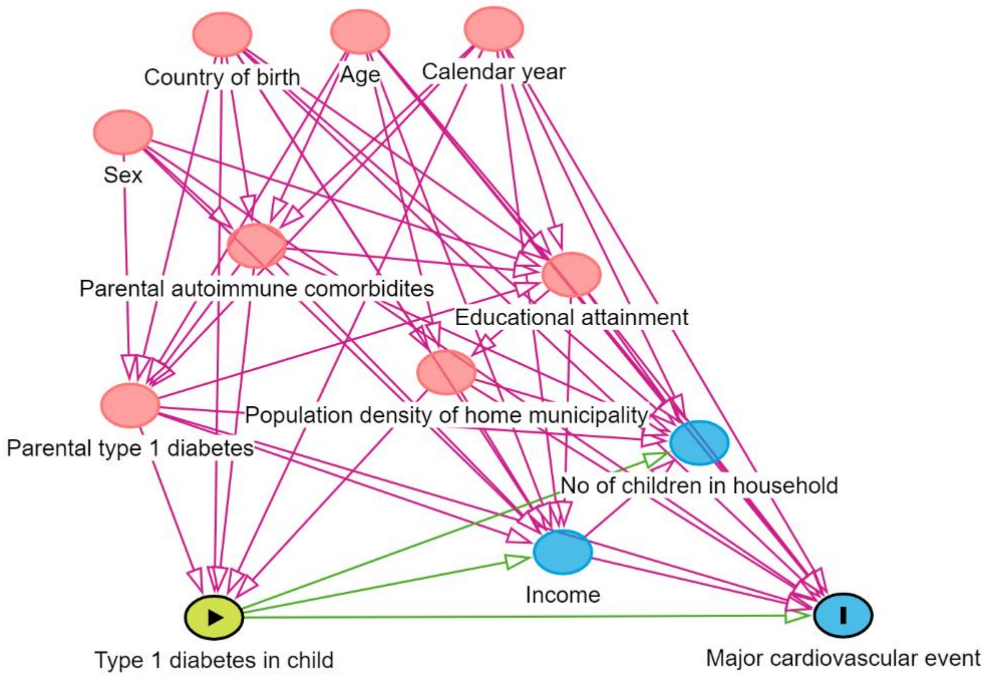

ESM Fig. 3. Time-varying coefficients for adjusted hazard ratios (aHRs) and 95% CIs for major cardiovascular events (MCE; acute myocardial infarction, ischaemic or haemorrhagic stroke, and cardiovascular death) and all-cause death in mothers and fathers of children with type 1 diabetes, as compared to population-based parental controls.

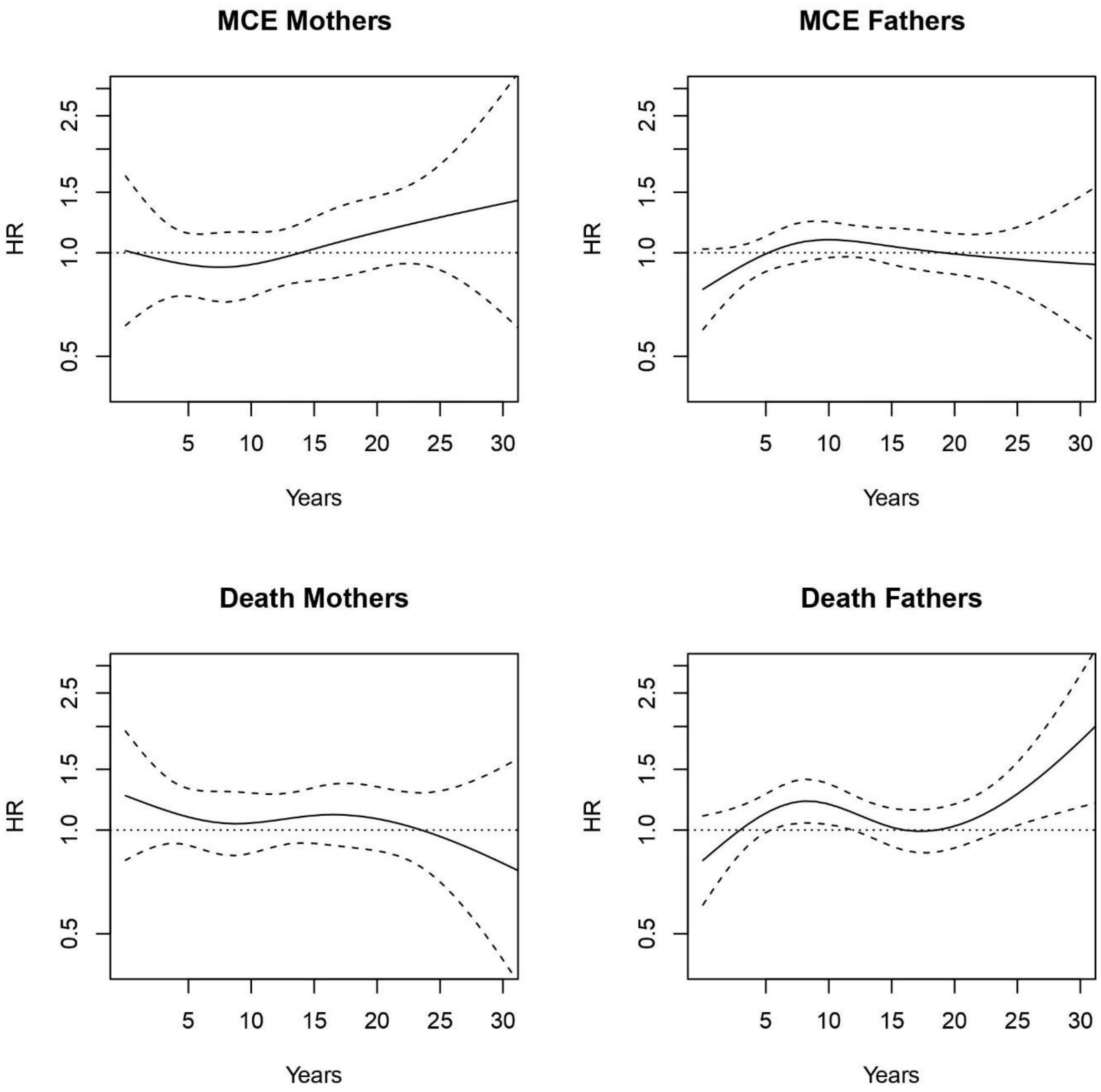

HRs adjusted for parental age (time-updated), type 1 diabetes status (time-updated), autoimmune comorbidities, country of birth and population density of home municipality.

REFERENCES

[1] SWEDIABKIDS Nationella Diabetesregistret - barn och ungdomsdiabetes www.ndr.nu (2020) Årsrapport: 2020 ÅRS RESULTAT.
